# Elevated Serum Trimethylamine N-Oxide Predicts Impaired Vascular Reactivity in Patients with Hypertension

**DOI:** 10.3390/diagnostics15182400

**Published:** 2025-09-20

**Authors:** I-Min Su, Ji-Hung Wang, Chin-Hung Liu, Bang-Gee Hsu

**Affiliations:** 1 Department of Anesthesiology, Dalin Tzu Chi Hospital, Buddhist Tzu Chi Medical Foundation, Chiayi 62247, Taiwan; 2 School of Medicine, Tzu Chi University, Hualien 97004, Taiwan; 3 Institute of Medical Sciences, Tzu Chi University, Hualien 97004, Taiwan; 4 Department of Internal Medicine, Hualien Tzu Chi Hospital, Buddhist Tzu Chi Medical Foundation, Hualien 97004, Taiwan; 5 Division of Cardiology, Buddhist Tzu Chi General Hospital, Hualien 97004, Taiwan; 6 Graduate Institute of Clinical Pharmacy, School of Medicine, Tzu Chi University, Hualien 97004, Taiwan; 7 School of Pharmacy, Tzu Chi University, Hualien 97004, Taiwan; 8 Division of Nephrology, Hualien Tzu Chi Hospital, Buddhist Tzu Chi Medical Foundation, Hualien 97004, Taiwan

**Keywords:** trimethylamine N-oxide, hypertension, vascular reactivity index, endothelial dysfunction, digital thermal monitoring

## Abstract

**Background/Objectives**: Trimethylamine N-oxide (TMAO), a gut microbiota-derived metabolite influenced by diet, has been linked to cardiovascular disease. Endothelial dysfunction, an early sign of vascular damage, is common in hypertension. This study examined the relationship between serum TMAO levels and endothelial function, assessed by the vascular reactivity index (VRI), in patients with hypertension. **Methods**: In total, 110 patients with hypertension were enrolled. Fasting serum TMAO was measured using high-performance liquid chromatography–mass spectrometry. Endothelial function was evaluated via digital thermal monitoring, with VRI categorized as good (>2.0), intermediate (1.0–1.9), or poor (<1.0). **Results**: Of the participants, 10 (9.1%) exhibited poor vascular reactivity, 57 (51.8%) had intermediate reactivity, and 43 (39.1%) exhibited good vascular reactivity. Poor reactivity correlated with older age (*p* = 0.010), higher total cholesterol (*p* = 0.007), low-density lipoprotein cholesterol (*p* = 0.009), and higher TMAO levels (*p* < 0.001). In multivariate forward stepwise linear regression, the log-transformed TMAO level (log-TMAO) remained independently and inversely associated with VRI (*p* < 0.001). Logistic regression analyses demonstrated that elevated TMAO concentrations were significantly associated with an increased likelihood of vascular reactivity dysfunction (intermediate and poor groups combined; odds ratio [OR] = 1.10, 95% confidence interval [CI]: 1.047–1.155; *p* < 0.001) and, in particular, with poor vascular reactivity (OR = 1.58, 95% CI: 1.002–2.492; *p* = 0.049). **Conclusions**: Elevated serum TMAO is independently associated with endothelial dysfunction in hypertension.

## 1. Introduction

Hypertension remains a significant global public health concern. According to the latest data from the National Health and Nutrition Examination Survey, the prevalence of hypertension among adults has reached 47.7% [1]. As a leading risk factor for cardiovascular diseases, its effective prevention and control are crucial for reducing premature cardiovascular mortality worldwide [2]. Blood pressure regulation is primarily determined by vascular dilation capacity and circulating intravascular fluid volume [3]. The vascular endothelium, positioned between the bloodstream and surrounding tissues, plays a pivotal role in cardiovascular homeostasis by regulating nutrient and metabolite exchange and mediating interactions with circulating cells, hormones, and cytokines [4]. Endothelial dysfunction—marked by impaired vasodilation, abnormal cell proliferation, increased platelet adhesion and activation, and pro-inflammatory, prothrombotic activity [5]—can precede the onset of clinical hypertension [6]. By promoting vasoconstriction and resistance artery remodeling through structural, mechanical, and functional changes, endothelial dysfunction contributes significantly to both the development and complications of hypertension [7].

Trimethylamine N-oxide (TMAO) is a small, colorless amine oxide produced through the microbial metabolism of dietary nutrients such as choline, betaine, and carnitine, which are abundant in foods like red meat, deep water fish, egg yolk, and certain dairy products. The resulting trimethylamine (TMA) is subsequently oxidized in the liver by flavin-containing monooxygenase 3 (FMO3) to form TMAO. Circulating TMAO levels are modulated by dietary intake, gut microbiota composition, and hepatic FMO3 activity [8,9]. Elevated plasma TMAO concentrations have been linked to a higher risk of major adverse cardiovascular events and mortality in humans [10,11]. Mechanistically, TMAO may impair endothelial function by reducing endothelial nitric oxide synthase (eNOS) phosphorylation and nitric oxide (NO) production, promoting the expression of adhesion molecules, and elevating pro-inflammatory cytokines, such as interleukin-6 [12,13,14]. Furthermore, TMAO contributes to atherosclerosis by disrupting cholesterol and sterol metabolism, promoting foam cell formation via upregulation of macrophage scavenger receptors, and altering bile acid metabolism and sterol transporter expression in the liver and intestines [15]. These mechanisms collectively promote endothelial dysfunction, thereby increasing the risk of cardiovascular complications [8].

Considering that endothelial dysfunction is both a consequence and an early marker of hypertensive vascular damage, identifying novel biomarkers such as TMAO may offer insights into early risk stratification and therapeutic targets in individuals with hypertension. Considering the potential role of TMAO in vascular dysfunction, this study aimed to investigate the relationship between serum TMAO levels and endothelial function, as assessed by the vascular reactivity index (VRI), in patients with hypertension.

## 2. Materials and Methods

### 2.1. Study Population

A total of 110 patients with hypertension were recruited from the cardiovascular outpatient clinic of Buddhist Tzu Chi General Hospital, Hualien, Taiwan, between December 2020 and July 2021. The study protocol was approved by the Research Ethics Committee of Hualien Tzu Chi Hospital, Buddhist Tzu Chi Medical Foundation (IRB108-219-A, 19 November 2019), and all participants provided written informed consent before enrollment. According to the JNC 8 criteria, hypertension was diagnosed when systolic blood pressure (SBP) was ≥140 mmHg, diastolic blood pressure (DBP) was ≥90 mmHg, or if antihypertensive drugs had been taken during the prior two weeks. Blood pressure was measured on the right arm using a standard mercury sphygmomanometer and appropriately sized cuffs after a 10 min rest in a seated position. Three readings were taken at 5 min intervals, and the average value was used for analysis. Diabetes mellitus (DM) was defined as a fasting plasma glucose ≥126 mg/dL or the use of oral hypoglycemic agents or insulin. Coronary artery disease (CAD) was diagnosed based on >50% stenosis in any coronary segment on angiography. The exclusion criteria included the presence of acute infections, malignancy, recent myocardial infarction, limb amputation, heart failure at the time of blood collection, or refusal to provide informed consent.

### 2.2. Anthropometric Measurements

In the morning after an overnight fast, participants underwent anthropometric evaluation. Height and weight were measured with a precision of 0.5 cm and 0.5 kg, respectively. Body mass index (BMI) was derived using the formula: weight (kg)/height^2^ (m^2^).

### 2.3. Biochemical Analysis

After overnight fasting, venous blood samples (approximately 5 mL) were collected into serum-separating tubes without anticoagulants. Samples were allowed to clot at room temperature for 30 min and then centrifuged at 3000× *g* for 10 min. The resulting serum was stored at 4 °C within 1 h of collection for biochemical testing and TMAO analysis. Biochemical parameters, including total calcium, phosphate, fasting glucose, albumin, globulin, blood urea nitrogen, creatinine, total cholesterol (TCH), triglycerides, high-density lipoprotein cholesterol (HDL-C), and low-density lipoprotein cholesterol (LDL-C), were assessed with the Siemens Advia 1800 autoanalyzer (Siemens Healthcare, Henkestr, Erlangen, Germany). Renal function was evaluated by calculating estimated glomerular filtration rate (eGFR) with the CKD-EPI (Chronic Kidney Disease Epidemiology Collaboration) formula.

### 2.4. Assessment of Endothelial Function

Endothelial function was assessed using a digital thermal monitoring system (VENDYS-II; Endothelix, Inc., Houston, TX, USA), approved by the U.S. Food and Drug Administration. Measurements were taken after an overnight fast and at least 12 h of abstinence from tobacco, alcohol, caffeine, and vasoactive medications. Participants rested in a supine position for 30 min in a room maintained at 22–24 °C. A blood pressure cuff was applied to the right upper arm, and temperature sensors were placed on the index fingers of both hands (left: control; right: occlusion). Thermal monitoring was performed during three phases: a 5 min baseline, 5 min cuff inflation to 50 mmHg above SBP, and a 5 min deflation to elicit reactive hyperemia. The VRI was automatically calculated by the VENDYS software (https://www.vendys2.com/ (accessed on 18 September 2025)) as the maximum temperature rebound from baseline and categorized as poor (<1.0), intermediate (1.0–1.9), or good (≥2.0) [16].

### 2.5. Serum TMAO Quantification by HPLC-MS

Serum TMAO concentrations were measured using a Waters e2695 HPLC system coupled to a single quadrupole mass spectrometer (ACQUITY QDa, Waters Corp., Milford, MA, USA). Separation was performed on a Phenomenex Luna^®^ C18(2) column (250 × 4.6 mm, 5 µm, 100 Å, Phenomenex, Torrance, CA, USA) at 40 °C with a binary gradient of water (0.1% formic acid) and methanol (0.1% formic acid) at a flow rate of 0.8 mL/min. The injection volume was 30 µL. Mass spectrometry was conducted in electrospray positive-ion mode with a desolvation temperature of 600 °C, capillary voltage of 0.8 kV, and cone voltage of 15 V. Data were acquired in single ion recording (SIR) mode, monitoring 76.0 *m*/*z* for TMAO and 85.1 *m*/*z* for the internal standard d9-TMAO. The retention time for TMAO and d9-TMAO was approximately 2.54 min [17]. Data acquisition and processing were performed using Empower^®^ 3.0 (Waters Corp., Milford, MA, USA). In our study, repeated measurements of quality control samples demonstrated an intra-assay coefficient of variation of 4.2% and an inter-assay coefficient of variation of 6.0% for TMAO detection. Calibration curves were constructed using serial dilutions of authentic TMAO standards (range starting from 1 μg/L), with correlation coefficients (R^2^) consistently >0.99.

### 2.6. Statistical Analysis

Data normality was examined using the Kolmogorov–Smirnov test. Continuous variables are reported as mean ± standard deviation when normally distributed, and as median with interquartile range when not. Group comparisons across VRI categories (poor, intermediate, good) were performed using the Jonckheere–Terpstra test for continuous variables and the Cochran–Armitage trend test for categorical variables. Variables including fasting glucose, triglycerides, BUN, creatinine, and TMAO were log-transformed to approximate normal distributions. Relationships between TMAO levels and vascular reactivity were analyzed using univariate and multivariate logistic regression models. Simple linear regression was used to assess correlations with VRI, followed by a forward stepwise multivariate regression for significant predictors. Associations between log-transformed TMAO and clinical parameters were assessed using nonparametric Spearman’s rank correlation. To evaluate the predictive performance of TMAO for vascular reactivity dysfunction and poor vascular reactivity, receiver operating characteristic (ROC) curve analysis was performed, and the corresponding AUC values were obtained (MedCalc v22.019, Ostend, Belgium). Statistical significance was defined as *p* < 0.05. Data analyses were carried out with SPSS version 19.0 (IBM Corp., Armonk, NY, USA).

## 3. Results

The clinical characteristics and patterns of antihypertensive medication use among the 110 enrolled hypertensive patients are presented in Table 1. Of these patients, 10 (9.1%) had poor VRI, 57 (51.8%) had intermediate VRI, and 43 (39.1%) had good VRI. Patients with lower VRI values were significantly older (*p* = 0.012) and had higher levels of TCH (*p* = 0.007), LDL-C (*p* = 0.009), and serum TMAO (*p* < 0.001). In terms of comorbidities, 51 patients (46.4%) had DM, 83 (75.5%) had CAD, and 20 (20.9%) were current smokers. No significant differences were found between VRI groups regarding sex, prevalence of DM or CAD, smoking status, or use of antihypertensive medications.

Logistic regression analysis demonstrated that higher serum TMAO levels were positively and independently associated with impaired vascular reactivity. Patients with poor or intermediate VRI exhibited a higher likelihood of vascular dysfunction (odds ratio [OR]: 1.100; 95% confidence interval [CI]: 1.047–1.155) than those with good VRI (*p* < 0.001). In the multivariate model, adjusted for age, sex, BMI, fasting glucose, eGFR, TCH, LDL-C, and TMAO, serum TMAO remained a significant predictor of poor VRI (OR: 1.580; 95% CI: 1.002–2.492; *p* = 0.049) (Table 2).

Table 3 presents the correlations between clinical parameters and VRI. VRI showed significant negative correlations with age (*r* = −0.233, *p* = 0.014), TCH (*r* = −0.229, *p* = 0.016), and log-transformed TMAO levels (log-TMAO, *r* = −0.530, *p* < 0.001). Stepwise linear regression identified log-TMAO as the strongest independent predictor of VRI (β = −0.530, adjusted R^2^ change = 0.274, *p* < 0.001).

**Figure 1:**
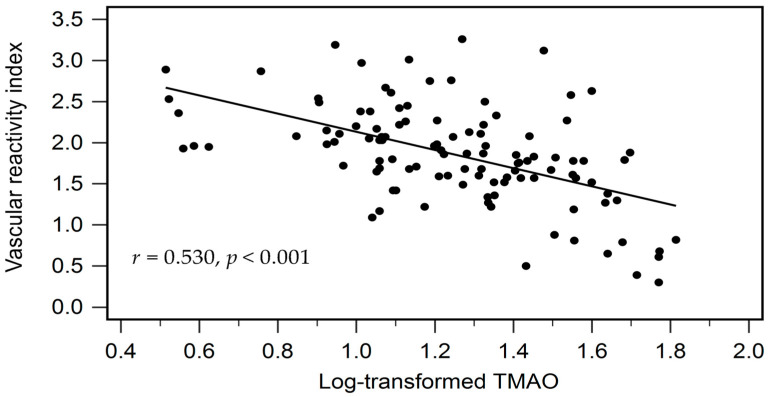
Associations between vascular reactive index (VRI) and log-transformed TMAO levels in hypertensive patients.

As shown in Table 4, Spearman correlation analysis demonstrated a significant negative correlation between serum log-TMAO levels and VRI (*r* = −0.530, *p* < 0.001). Furthermore, log-TMAO was positively correlated with age (*r* = 0.191, *p* = 0.046) and negatively correlated with eGFR (*r* = −0.246, *p* = 0.010). Figure 1 displays the two-dimensional scattered plots of VRI values in relation to serum log-TMAO levels in hypertensive patients.

The ROC analysis showed that serum TMAO levels predicted vascular reactivity dysfunction with an AUC of 0.770 (95% CI: 0.681–0.859, *p* < 0.001), and poor vascular reactivity with an AUC of 0.950 (95% CI: 0.898–1.000, *p* < 0.001). Based on the Youden index, the optimal cutoff value of TMAO for predicting vascular reactivity dysfunction was 13.62 μg/L, yielding a sensitivity of 80.6%, specificity of 65.12%, positive predictive value of 78.3%, and negative predictive value of 68.3%. For poor vascular reactivity, the optimal cutoff was 26.23 μg/L, with a sensitivity of 100.0%, specificity of 79.0%, positive predictive value of 32.3%, and negative predictive value of 100.0% (Table 5).

## 4. Discussion

In this study of patients with hypertension, higher serum TMAO levels were strongly associated with impaired endothelial function, as measured by VRI. Individuals with lower VRI values tended to have higher serum TMAO, TCH, and LDL-C levels and were older. Importantly, both univariate and multivariate logistic regression analyses demonstrated that serum TMAO levels were independently associated with the risk of vascular reactivity dysfunction. Furthermore, TMAO demonstrated a robust negative correlation with VRI and remained a significant predictor in stepwise linear regression analysis. These findings suggest that TMAO is a promising biomarker of endothelial dysfunction in patients with hypertension, highlighting a potential mechanistic connection between gut microbiota–derived metabolites and vascular health in this population. Consistent with our results, Chen et al. reported that serum adipocyte fatty acid-binding protein levels were independently and inversely correlated with VRI in kidney transplant patients [18]. Together, these findings suggest that distinct classes of metabolites—including adipokines and gut microbiota-derived metabolites—may converge on common endothelial pathways that impair vascular reactivity.

The vascular endothelium is essential for maintaining vascular homeostasis, primarily through the release of signaling molecules that regulate vasomotor tone, blood fluidity, immune function, and vascular permeability. These processes rely on a delicate balance between vasodilators and vasoconstrictors, antioxidants and oxidants, and pro- and anti-inflammatory mediators [19,20]. Disruption of this equilibrium leads to endothelial dysfunction, characterized by heightened vascular tone, inflammation, thrombosis, and oxidative stress [21]. Advancing age is among the most significant independent predictors of endothelial impairment. Aging contributes to progressive vascular stiffening and reduced nitric oxide (NO) bioavailability, both of which diminish endothelium-dependent vasodilation in large and small arteries [22]. Consistent with prior research—including findings by Naghavi et al., which demonstrated an inverse relationship between VRI and age in a large U.S. outpatient cohort [16]—our study likewise found that patients with lower VRI values were significantly older. Moreover, dyslipidemia—particularly elevated TCH and LDL-C levels—is a well-established contributor to endothelial dysfunction and cardiovascular risk [23]. Oxidized LDL-C impairs endothelial function by reducing eNOS activity and NO production, while simultaneously promoting macrophage activation and foam cell formation, thus accelerating atherogenesis [24,25,26]. In agreement with previous findings [27], our results show that patients with lower VRI values—indicative of impaired endothelial function—had significantly higher TCH and LDL-C levels, further underscoring the link between lipid abnormalities and vascular health.

Beyond traditional risk factors such as age and dyslipidemia, emerging evidence has identified gut microbiota-derived metabolites—particularly TMAO—as key contributors to the development of endothelial dysfunction. TMAO is produced through a two-step process: gut microbes convert dietary precursors such as choline and carnitine into trimethylamine (TMA), which is then oxidized in the liver to TMAO by flavin-containing monooxygenase 3 (FMO3) [8,28]. Brunt et al. reported that healthy middle-aged and older adults (≥50 years) exhibit significantly higher plasma TMAO levels than younger individuals, independent of dietary intake of TMAO precursors [11]. This increase is likely driven by age-related changes in gut microbiota composition—particularly a greater abundance of TMA-producing genera such as *Desulfovibrio*—as well as increased hepatic FMO3 expression [29]. Moreover, because TMAO is primarily cleared by the kidneys, age-associated declines in renal function can further promote its accumulation in the circulation [30]. In line with these mechanisms, our study found that higher plasma TMAO concentrations were significantly associated with older age and lower eGFR. These results support the hypothesis that aging and renal impairment may act synergistically to raise TMAO levels, thereby contributing to vascular dysfunction through nontraditional pathways.

Expanding upon the associations identified with traditional risk factors, our study underscores a strong and independent link between circulating TMAO levels and endothelial dysfunction, as measured by VRI. Multivariate regression analyses revealed that higher serum log-TMAO remained a significant negative predictor of VRI, even after adjusting for potential confounders such as age, lipid profile, renal function, and glycemia. Logistic regression further confirmed that elevated TMAO levels independently increased the likelihood of vascular reactivity dysfunction. These results align with growing evidence that TMAO is not merely a marker of vascular injury but may actively contribute to endothelial impairment [31]. For instance, Zhou et al. reported that elevated TMAO levels are correlated with lower endothelial progenitor cells and reduced flow-mediated dilation, reflecting peripheral endothelial dysfunction in human subjects, and that direct exposure to TMAO can impair endothelial progenitor cells’ function in vitro [32]. Both preclinical and clinical studies indicate that TMAO disrupts endothelial function by decreasing eNOS phosphorylation, inhibiting nitric oxide synthesis, enhancing oxidative stress, increasing adhesion molecule expression, and inducing pro-inflammatory cytokine release [16]. Mechanistic studies have further implicated TMAO in activating the NOD-like receptor family pyrin domain-containing 3 (NLRP3) inflammasome and Cathepsin B pathways, which promote cytokine release and compromise endothelial junction integrity, thereby impairing vascular reactivity [33]. Consistent with these mechanistic insights, our finding that TMAO is a strong, independent correlate of reduced VRI provides clinical evidence supporting its contributory role in endothelial dysfunction, particularly among individuals with hypertension.

This study has several limitations that warrant consideration. First, its cross-sectional design prevents establishing a causal relationship between serum TMAO levels and vascular reactivity. Longitudinal investigations are needed to clarify whether elevated TMAO levels drive the progression of endothelial dysfunction over time. Second, the study cohort consisted exclusively of patients with hypertension from a single center, which may restrict the applicability of our findings to broader or more diverse populations. Third, although multiple potential confounders were accounted for, residual confounding from unmeasured factors—such as dietary patterns, gut microbiota composition, or inflammatory status—cannot be entirely excluded. Fourth, TMAO measurements were obtained at a single time point, which may not fully capture long-term exposure or temporal variability. Fifth, our study cohort consisted predominantly of male participants (87.3%), which may affect the representativeness of the results and limit the extrapolation to the general hypertensive population, particularly to females who may exhibit different vascular characteristics. Lastly, the relatively small sample size of the poor VRI group (*n* = 10) may reduce the statistical power of the analyses and the robustness of the findings. Larger, multicenter studies with more balanced sex distribution and greater representation across VRI categories are warranted to validate our observations and enhance their generalizability.

## 5. Conclusions

Our findings reveal a strong and independent relationship between elevated serum TMAO levels and impaired endothelial function in patients with hypertension. These results highlight TMAO’s potential as a noninvasive biomarker for early detection of vascular dysfunction and suggest that interventions targeting TMAO-related pathways could offer a novel strategy for cardiovascular risk assessment and management in hypertensive populations.

## Figures and Tables

**Table 1:** Clinical characteristics according to different vascular reactivity indices by digital thermal monitoring of the 110 hypertensive patients.

Characteristics	All Participants (*n* = 110)	Good Vascular Reactivity (*n* = 43)	Intermediate Vascular Reactivity (*n* = 57)	Poor Vascular Reactivity (*n* = 10)	*p* Value
Age (years)	63.29 ± 8.43	61.98 ± 7.61	63.18 ± 8.87	69.61 ± 6.87	0.010 *
Height (cm)	164.96 ± 6.85	164.12 ± 7.00	165.45 ± 6.98	165.80 ± 5.43	0.487
Body weight (kg)	72.22 ± 11.79	71.42 ± 9.52	73.26 ± 13.45	69.77 ± 10.95	0.693
Body mass index (kg/m^2^)	26.47 ± 3.59	26.50 ± 3.13	26.66 ± 4.00	25.27 ± 2.99	0.330
Vascular reactivity index	1.86 ± 0.61	2.43 ± 0.35	1.64 ± 0.24	0.64 ± 0.19	<0.001 *
Systolic BP (mmHg)	136.95 ± 18.28	138.33 ± 18.14	135.58 ± 18.28	138.80 ± 20.13	0.942
Diastolic BP (mmHg)	80.31 ± 11.27	82.16 ± 10.86	79.28 ± 11.91	78.20 ± 8.75	0.319
Total cholesterol (mg/dL)	161.88 ± 39.29	159.95 ± 35.17	157.25 ± 37.02	196.60 ± 53.88	0.007 *
Triglyceride (mg/dL)	131.50 (103.50–188.50)	122.00 (102.00–184.00)	131.00 (103.00–194.50)	153.50 (101.50–201.75)	0.926
HDL-C (mg/dL)	46.51 ± 11.85	47.51 ± 9.24	45.84 ± 14.18	46.00 ± 6.63	0.719
LDL-C (mg/dL)	90.44 ± 30.77	90.56 ± 28.29	85.49 ± 26.91	118.10 ± 47.15	0.009 *
Fasting glucose (mg/dL)	109.00 (91.00–141.50)	114.00 (91.00–139.00)	105.00 (91.50–148.00)	97.00 (77.00–136.75)	0.321
Blood urea nitrogen (mg/dL)	17.00 (14.00–20.00)	16.00 (14.00–18.00)	17.00 (14.00–22.00)	17.50 (12.75–22.75)	0.343
Creatinine (mg/dL)	1.00 (0.90–1.10)	1.00 (0.80–1.10)	1.00 (0.90–1.20)	1.00 (0.90–1.05)	0.385
eGFR (mL/min)	77.47 ± 18.19	79.99 ± 16.21	75.34 ± 19.08	78.75 ± 21.35	0.846
TMAO (μg/L)	18.65 (11.45–28.34)	11.86 (9.05–18.61)	21.33 (13.92–28.35)	49.73 (34.91–58.97)	<0.001 *
Male, *n* (%)	96 (87.3)	37 (86.0)	50 (87.7)	9 (90.0)	0.935
Diabetes mellitus, *n* (%)	51 (46.4)	21 (48.8)	24 (42.1)	6 (60.0)	0.530
Coronary artery disease, *n* (%)	83 (75.5)	35 (81.4)	40 (70.2)	8 (80.0)	0.409
Smoking, *n* (%)	23 (20.9)	12 (27.9)	8 (14.0)	3 (30.0)	0.183
ACE inhibitor use, *n* (%)	20 (18.2)	8 (18.6)	11 (19.3)	1 (10.0)	0.778
ARB use, *n* (%)	51 (46.4)	23 (53.5)	24 (42.1)	4 (40.0)	0.483
β-blocker use, *n* (%)	55 (50.0)	19 (44.2)	29 (50.9)	7 (70.0)	0.333
CCB use, *n* (%)	44 (40.0)	20 (46.5)	19 (33.3)	5 (50.0)	0.328
Statin use, *n* (%)	87 (79.1)	30 (69.8)	49 (86.0)	8 (80.0)	0.143
Fibrate use, *n* (%)	6 (5.5)	3 (7.0)	2 (3.5)	1 (10.0)	0.603

Data are presented as mean ± standard deviation for continuous variables with normal distribution (analyzed by one-way ANOVA) and as median with interquartile range for skewed data (tested with the Jonckheere–Terpstra test). Categorical variables are expressed as *n* (%) and analyzed with the Cochran–Armitage trend test. Abbreviations: BP = blood pressure; HDL-C = high-density lipoprotein cholesterol; LDL-C = low-density lipoprotein cholesterol; eGFR = estimated glomerular filtration rate; TMAO = trimethylamine N-oxide; ACE = angiotensin-converting enzyme; ARB = angiotensin receptor blocker; CCB = calcium channel blocker. * *p* < 0.05 was considered statistically significant.

**Table 2:** Multivariate logistic regression analysis for vascular reactivity dysfunction (intermediate vascular reactivity and poor vascular reactivity) or poor vascular reactivity in 110 hypertensive patients.

Model	TMAO (per 1 μg/L of Increase) for Vascular Reactivity Dysfunction		TMAO (per 1 μg/L of Increase) for Poor Vascular Reactivity	
	OR (95% CI)	*p* Value	OR (95% CI)	*p* Value
Crude model	1.098 (1.047–1.150)	<0.001 *	1.176 (1.084–1.275)	<0.001 *
Model 1	1.100 (1.048–1.154)	<0.001 *	1.174 (1.079–1.278)	<0.001 *
Model 2	1.100 (1.047–1.155)	<0.001 *	1.580 (1.002–2.492)	0.049 *

Model 1: adjusted for age, sex, and body mass index. Model 2: adjusted for Model 1 plus fasting glucose, eGFR, total cholesterol, and LDL-C. Abbreviations: LDL-C = low-density lipoprotein cholesterol; eGFR = estimated glomerular filtration rate; TMAO = trimethylamine N-oxide; OR = odds ratio; CI = confidence interval. * *p* < 0.05 was considered statistically significant.

**Table 3:** Correlation of vascular reactivity index levels and clinical variables by simple or multivariable linear regression analyses among 110 hypertensive patients.

Variables	Vascular Reactivity Index				
	Simple Regression		Multivariable Regression		
	*r*	*p* Value	Beta	Adjusted *r*^2^ Change	*p* Value
Age (years)	−0.233	0.014 *	–	–	–
Height (cm)	−0.085	0.375	–	–	–
Body weight (kg)	−0.028	0.768	–	–	–
Body mass index (kg/m^2^)	0.027	0.778	–	–	–
Systolic blood pressure (mmHg)	0.038	0.692	–	–	–
Diastolic blood pressure (mmHg)	0.154	0.109	–	–	–
Total cholesterol (mg/dL)	−0.229	0.016 *	–	–	–
Log-Triglyceride (mg/dL)	−0.031	0.748	–	–	–
HDL-C (mg/dL)	0.059	0.544	–	–	–
LDL-C (mg/dL)	−0.176	0.065	–	–	–
Log-Glucose (mg/dL)	0.162	0.091	–	–	–
Log-BUN (mg/dL)	−0.152	0.112	–	–	–
Log-Creatinine (mg/dL)	−0.094	0.329	–	–	–
eGFR (mL/min)	0.089	0.357	–	–	–
Log-TMAO (μg/L)	−0.530	<0.001 *	−0.530	0.274	<0.001 *

Variables with skewed distribution (triglyceride, fasting glucose, BUN, creatinine, and TMAO) were log-transformed prior to regression analyses. Simple and multivariate stepwise linear regression (adjusted for age, total cholesterol, and log-TMAO) were performed. Abbreviations: HDL-C = high-density lipoprotein cholesterol; LDL-C = low-density lipoprotein cholesterol; BUN = blood urea nitrogen; eGFR = estimated glomerular filtration rate; TMAO = trimethylamine N-oxide. * *p* < 0.05 was considered statistically significant.

**Table 4:** Spearman correlation coefficients between serum log-transformed trimethylamine N-oxide levels and clinical variables in 110 hypertensive patients.

Variables	Spearman Coefficient of Correlation	*p* Value
Age (years)	0.191	0.046 *
Body mass index (kg/m^2^)	−0.066	0.495
Vascular reactivity index	−0.530	<0.001 *
Systolic blood pressure (mmHg)	−0.077	0.427
Diastolic blood pressure (mmHg)	−0.130	0.176
Total cholesterol (mg/dL)	0.145	0.130
Log-Triglyceride (mg/dL)	−0.035	0.714
HDL-C (mg/dL)	−0.047	0.624
LDL-C (mg/dL)	0.060	0.531
Log-Glucose (mg/dL)	−0.048	0.620
eGFR (mL/min)	−0.246	0.010 *

Triglyceride, glucose, and TMAO data showed skewed distributions and were log-transformed before statistical testing. Spearman correlation analysis was used to evaluate associations. Abbreviations: HDL-C = high-density lipoprotein cholesterol; LDL-C = low-density lipoprotein cholesterol; eGFR = estimated glomerular filtration rate. * *p* < 0.05 was considered statistically significant (two-tailed).

**Table 5:** Diagnostic value of trimethylamine N-oxide levels on vascular reactivity dysfunction (intermediate vascular reactivity and poor vascular reactivity) or poor vascular reactivity.

	Vascular Reactivity Dysfunction						
	AUC (95% CI)	*p* Value	Cut-Off	Sen (%)	Spe (%)	PPV (%)	NPV (%)
TMAO (μg/L)	0.770 (0.681–0.859)	*p* < 0.001 *	13.62	80.60	65.12	78.26	68.30
	**Poor Vascular Reactivity**						
	**AUC (95% CI)**	***p*** **Value**	**Cut-Off**	**Sen (%)**	**Spe (%)**	**PPV (%)**	**NPV (%)**
TMAO (μg/L)	0.950 (0.898–1.000)	*p* < 0.001 *	26.23	100.0	79.00	32.26	100.0

Abbreviations: TMAO = trimethylamine N-oxide; AUC = area under the curve; 95% CI = 95% confidence interval; Sen = sensitivity; Spe = specificity; PPV = positive predictive value; NPV = negative predictive value. * *p* < 0.05 was considered statistically significant.

## Data Availability

The data presented in this study are available on reasonable request from the corresponding author. The data are not publicly available due to ethical restrictions and institutional policies protecting participant privacy (IRB108-219-A).

## Abbreviations

The following abbreviations are used in this manuscript:

BMI	Body mass index
CAD	Coronary artery disease
CKD-EPI	Chronic Kidney Disease Epidemiology Collaboration
DBP	Diastolic blood pressure
DM	Diabetes mellitus
eGFR	Estimated glomerular filtration rate
eNOS	Endothelial nitric oxide synthase
FMO3	Flavin-containing monooxygenase 3
HDL-C	High-density lipoprotein cholesterol
HPLC-MS	High-performance liquid chromatography-mass spectrometry
LDL-C	Low-density lipoprotein cholesterol
NLRP3	NOD-like receptor family pyrin domain containing 3
NO	Nitric oxide
SBP	Systolic blood pressure
TCH	Total cholesterol
TMA	Trimethylamine
TMAO	Trimethylamine N-oxide
VRI	Vascular reactivity index
